# Interacting Factors Associated with Adult Male Drowning in New Zealand

**DOI:** 10.1371/journal.pone.0130545

**Published:** 2015-06-17

**Authors:** James L. Croft, Chris Button

**Affiliations:** 1 Centre for Exercise and Sports Science Research, School of Exercise and Health Sciences, Edith Cowan University, Joondalup, Western Australia, Australia; 2 School of Physical Education, Sport and Exercise Sciences, University of Otago, Dunedin, New Zealand; Örebro University, SWEDEN

## Abstract

**Objectives:**

i) to identify factors that contribute to the global trend of the higher incidence of male drowning relative to females, and; ii) to explore relationships between such factors from mortality data in New Zealand.

**Methods:**

Drownings from 1983 to 2012 were examined for: *Age*, *Ethnicity*, *Site*, *Activity*, *Buoyancy and Alcohol*. Conditional frequency tables presented as mosaic plots were used to assess the interactions of these factors.

**Results:**

Alcohol was involved in a high proportion of *Accidental Immersion* drownings (61%) and was highest for males aged 20-24 years. When alcohol was involved there were proportionally more incidences where a life jacket was *Available But Not Worn* and less incidences where a life jacket was *Worn*. Many 30-39 year old males drowned during underwater activities (e.g., snorkeling, diving). Older men (aged +55 years old) had a high incidence of drowning while boating. Different ethnicities were over-represented in different age groups (Asian men aged 25-29, and European men aged 65-74) and when involved in different activities.

**Conclusions:**

Numerous interacting factors are responsible for male drownings. In New Zealand, drowning locations and activities differ by age and ethnicity which require targeted intervention strategies.

## Introduction

There is a strong global trend showing that males are at higher risk of drowning than females [1]. Data from the Global Burden of Disease analysis in 2000 indicates that a staggering 97% of drowning occurs in low and middle-income countries with the Western Pacific region accounting for 38%. In such countries the factors associated with male drowning may be quite different to those present in countries like New Zealand, Australia and the USA. In developed countries, the focus of this research, the ratio of male to female drowning varies depending on many factors such as age, location and socio-economic status with a range from two and a half to 12 times as many males compared to females (e.g., Australia [2]; UK [3]; US [4]; and comparison by socio-economic status: [5]). The higher risk factors for males seem to be replicated in most, if not all developed countries, including New Zealand [6]. Potential explanations that have been suggested for the greater number of male drownings include: increased exposure to risky situations; underestimation of risk and overestimation of ability; alcohol usage and associated decision making when under the influence of alcohol.

One common hypothesis offered regarding the difference in drowning rates between males and females is that males have a greater exposure to aquatic environments and to high-risk activities [7]. Compared with women, men spend significantly more time involved in aquatic activities. One study reported that males spent 61 days in the previous year involved in some sort of aquatic activity whereas females spent 52 days (p = 0.02) [7]. However, even when accounting for higher exposure to water-related activities in males, the ratio of drowning deaths to the number of water-related activities is still over three times higher for males than for females [8]. Hence increased exposure alone does not totally explain the greater risk of drowning in males. Clearly males are exposed to risk in aquatic environments more than females but equally important is that they typically behave in different ways around water.

In general, risks tend to be judged as lower by men than by women [9,10]. Some studies [7,11,12] have identified a propensity among adult males to underestimate the risk of drowning, and to overestimate their ability to cope with the risk inherent in water-related activity. Moran [13] found that 40% of male youth rated being caught in a rip current at a surf beach as only “slight/no risk” and 23% thought the risk of being swept off isolated rocks by a wave when fishing was “slight/no risk”. Self-reported confidence is also generally reported as greater in men than women. People with more water confidence (measured by their perceived anxiety) are more likely to engage in water-related activities in unsafe water locations than those with less confidence [14]. In aquatic contexts unsafe behaviors may be acts of independence and the capacity to take and manage risks themselves. For example, male youths are more likely than females to ignore safety directions, swim alone, dive into unknown depth, swim after alcohol or drugs, swim in prohibited areas, swim when cold or tired, and swim outside patrolled areas [15].

Underestimating the risk and overestimating the ability to cope may partially explain the high drowning rate in males. However, other socio-cultural practices and ethnicity linked variables may also contribute to the increased likelihood. Males may engage in risky aquatic activities that have some cultural significance. For example, gathering traditional sources of food, such as shellfish is part of Māori culture. Gathering adequate quantities of kaimoana (food from the sea) is now more difficult due to decreased availability. Using traditional methods such as diving may require deeper dives than previously, which increases the risk of drowning. Furthermore immigrant ethnicities may be over-represented in drowning statistics. A study in the United States found that “Recent immigrants (first or second generation) were found to be most at risk because they are most likely to practice seafood collection, preparation, and consumption habits closely resembling those in their native countries” [16].

Another factor that can contribute to poor decision-making is alcohol. Alcohol levels in global drowning fatalities from recreational swimming and boating were recently reviewed [17]. Alcohol was detected in 30–70% of people who drown while involved in recreational aquatic activity and probably contributes to death in at least 10–30% of cases. Blood alcohol level is not routinely documented in drowning victims in New Zealand and witness statements probably under report the usage of alcohol due to the negative societal and legal implications of alcohol impairment. A study that tried to estimate the magnitude of the effect [18], found that between 30–40% of drowning deaths had a positive blood alcohol content, which is similar to other developed countries and much greater than the reported national figure of 16%. A similar scenario may occur in Australia where alcohol involvement was present in 22% of drowning deaths (2002–2007) but was not reported for 35% of cases [19].

In summary, males typically spend more time in water-related activities than females although the increased exposure alone does not completely explain the increased risk of drowning. Males generally report more confidence in water than females and engage in riskier activities (i.e., swimming alone, in non-regulated locations). Males typically underestimate risk and overestimate their ability to cope. Alcohol is involved in 30–70% of people who drown while involved in recreational aquatic activity and probably contributes to death in 10–30% of cases. Excessive alcohol usage is likely to contribute to poor risk assessment and decision-making in recreational aquatic activity.

New Zealand has a long coastline and many rivers and lakes. Access to the water is common for a lot of the relatively small population. Boat ownership rates are some of the highest in the world and yet the laws regulating boating are less strict than in other countries. In general, New Zealanders feel connected with the land and the ocean, and fishing and water-based recreation is common. A large part of the population descends from Māori and Pacific island people, for whom fishing and gathering shellfish is part of their heritage. The combination of relative affluence and cultural beliefs creates potential drowning situations that may be different to other developed countries.

The relative role of factors that contribute to drowning risk in New Zealand is complex and not well understood. Further analysis of the interaction of different factors influencing the prevalence of male drowning is required. By considering the interaction of several key factors for male drowning through conditional frequency tables we illustrate a potentially valuable analysis procedure for epidemiological data.

## Methods

Male drowning data from 1980 to 2012 were retrieved from Water Safety New Zealand’s Drownbase, which is a database containing coroner report information from incidents in New Zealand or in its surrounding waters. Permission to access and subsequently publish mortality data was solicited from Water Safety New Zealand. All identifying data was removed from the data before we received it and individual cases were coded numerically. From 3,699 initial cases the following conditions were removed sequentially: Non-residents (n = 310); Missing the *Age* variable (n = 15); classified as *Commercial* (n = 22); *Suicides* in *Medical Condition* or *Activity* (n = 341); involved a “Vehicle” (n = 362); Commercial Fishing (n = 174). Further, two records were removed because they had too many missing fields and all children aged under 15 were removed. Drugs were only involved in 20 cases and were removed. Finally, the last 30 years (1983–2012) were selected leaving 2,134 cases for analysis.

Five-year age categories were created from the *Age* variable starting from 15–19 up to 75–79 with all those over 80 categorized together. All analysis was done using the R programming language [20].

**Fig 1:**
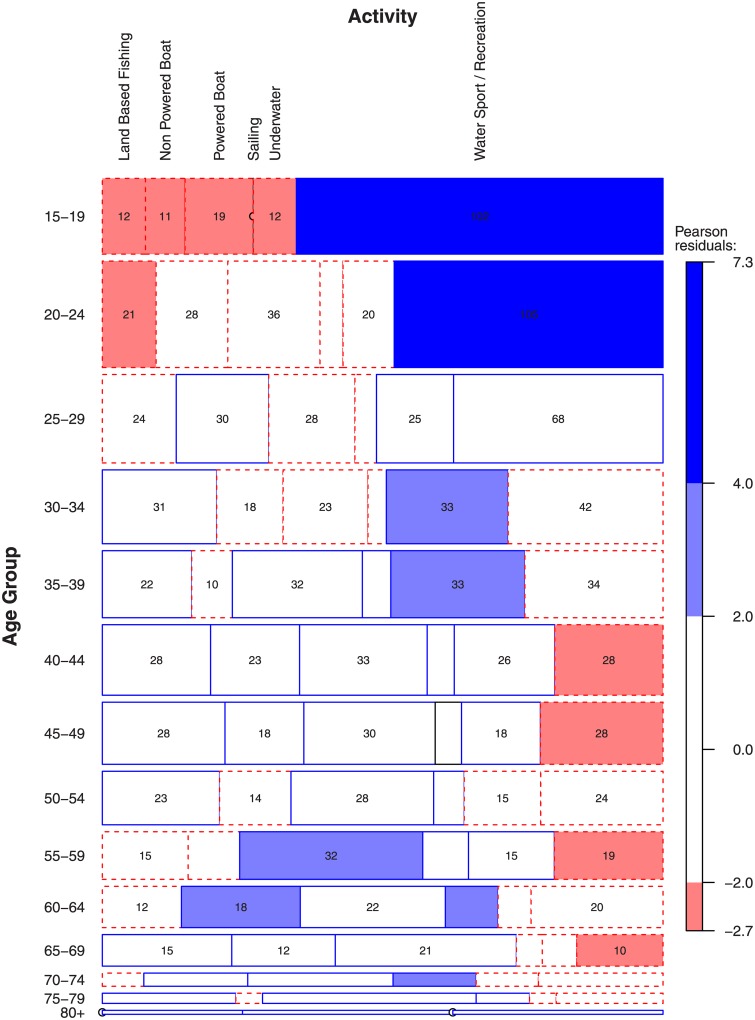
Age group affects the type of activity that precedes drowning. The size of a cell represents the frequency of drowning deaths given the factors listed around the edges of the plot (e.g., Activity and Age Group when alcohol was/was not involved). The boundary line of the cells indicate whether the cell is larger (solid line) or smaller (dashed line) than expected if the factors were independent. The size of residuals determines the lightness of a cell: very dark grey for large residuals (> 4, corresponding to α = 0.0001), lighter grey for medium sized residuals (< 4 and > 2), white for small residuals (< 2, corresponding to α = 0.05). For example, the data for 15–19 year olds under the Water Sport / Recreation category has a colour intensity greater than 4 and a boundary line. This indicates that the proportion of cases in this category is greater than expected for this age group.

We investigated the interaction of several key factors identified in the public health literature (i.e., Age, Activity, Site, Ethnicity, Alcohol, Bouyancy Aid) using conditional frequency tables that are presented graphically as mosaic plots [21]. These graphics can be thought of as summarizing the influence of interacting variables, such as the number of men drowned in situation X while doing activity Y while wearing/not wearing a bouyancy aid. In each plot, the size of any cell represents the frequency of drowning deaths given the factors listed around the edges of the plot (e.g., Activity and Age Group in Fig 1). Where more than ten or fifteen (depending on the size of the cells) drownings occurred the frequency is also shown within the cell. The boundary line of the cells indicate whether the cell is larger (solid line) or smaller (dotted line) than expected if the factors were independent [22]. The size of residuals determines the tone of a cell: very dark for large residuals (> 4), less dark for medium sized residuals (< 4 and > 2), white for small residuals (< 2). Cut-offs are usually chosen as 2 and 4 because Pearson residuals are normally distributed which implies that cells with these residuals are individually significant at approximately α = 0.05 and α = 0.0001 levels. The overall *p*-value is calculated from a χ^2^ distribution with the appropriate number of degrees of freedom.

## Results

The groupings of dark blue cells with solid boundary lines in Fig 1 show three clusters where certain activities were over represented within certain age groups: (1) 15–24 year olds drowned more than expected during Water Sport / Recreation (15–19, N = 102/156, 65%; 20–24, N = 105/219, 48%); (2) a high proportion of 30–39 year olds drowned during underwater activities (N = 66/290, 22%); (3) men aged 55–64 had a high incidence of drowning while boating (55–59, Powered boat N = 32/98, 33%; 60–64, Non-powered boat N = 18/85, 21%).

**Fig 2:**
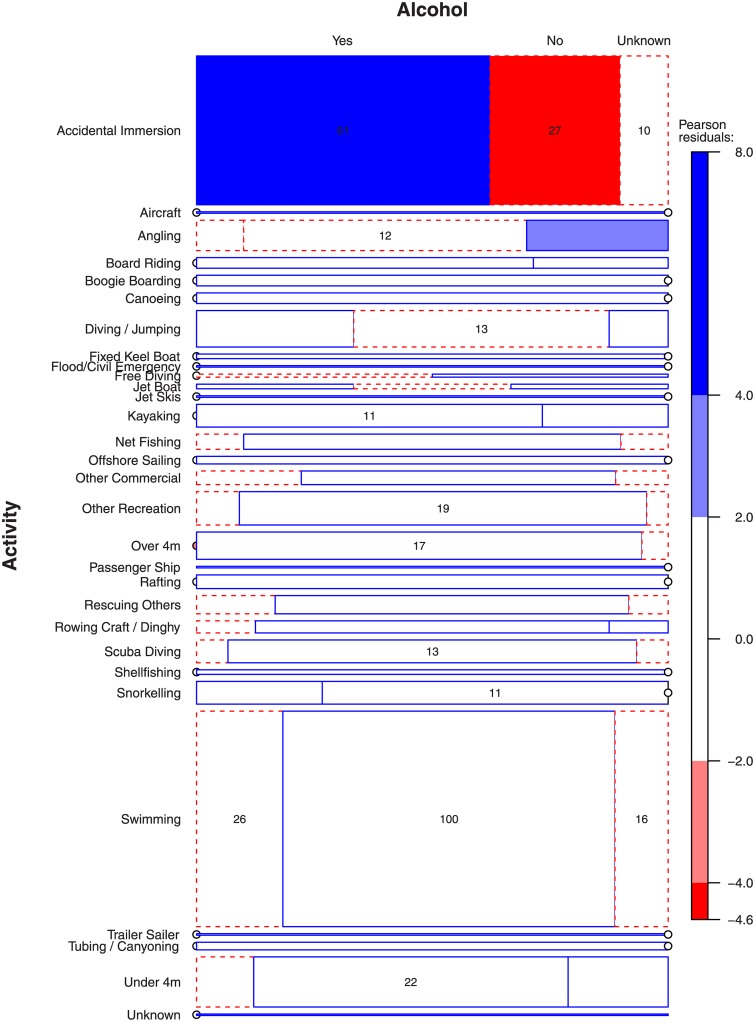
Most drownings occur following Accidental Immersions (i.e. falling in) and Swimming. *Over 4m* refers to powerboats over 4 m in length, *Under 4m* refers to powerboats under 4 m in length.

We investigated each of these groups further by looking at the interaction of a more detailed level of activity and the incidence of alcohol use (Fig 2). The highest frequency activities were ‘Accidental Immersion’ (N = 98) and ‘Swimming’ (N = 142), signified by the large area of those bars. A high proportion (62%) of accidental immersion drownings involved alcohol but the remaining activities were not significantly over represented by alcohol involvement. Young New Zealand males (15–24 years old) figure prominently in drownings associated with Water Sport / Recreation activities (15–19, N = 102 of 156 cases in that age group for all activities; 20–24, N = 105/219). Indeed, the high proportion of Water Sport / Recreation drownings is also present in the 25–29 age group with 68 cases (of 182 cases in that age group for all activities) but this trend didn’t quite reach significance. The highest frequency activities preceding drowning were recorded as ‘Accidental Immersion’ and ‘Swimming’. Of the underwater drownings in males aged 30–39 most occurred in Scuba Diving (Table 1). Not surprisingly, alcohol was not involved in many underwater deaths. Of males aged 55–64 who drowned while boating (Non-powered boat and Powered boat), alcohol was involved in a high proportion of Rowing Craft / Dinghy incidences (Table 2) and much less frequently in all other boat types.

**Table 1:** Activities of males aged 30–39 who drowned in Underwater activities and the involvement of alcohol.

	Alcohol		
Activity	Yes	No	Unknown
**Free Diving**	0	3	1
**Scuba Diving**	6	34	4
**Snorkeling**	1	16	1

**Table 2:** Activities of males aged 55–64 who drowned in while boating (Non powered boat and Powered boat) and the involvement of alcohol.

	Alcohol		
Activity	Yes	No	Unknown
**Canoeing**	0	1	0
**Jet Boat**	0	1	0
**Kayaking**	0	2	0
**Powerboat over 4m**	3	18	8
**Rafting**	0	1	0
**Rowing Craft / Dinghy**	9	11	3
**Powerboat under 4m**	2	16	6

**Fig 3:**
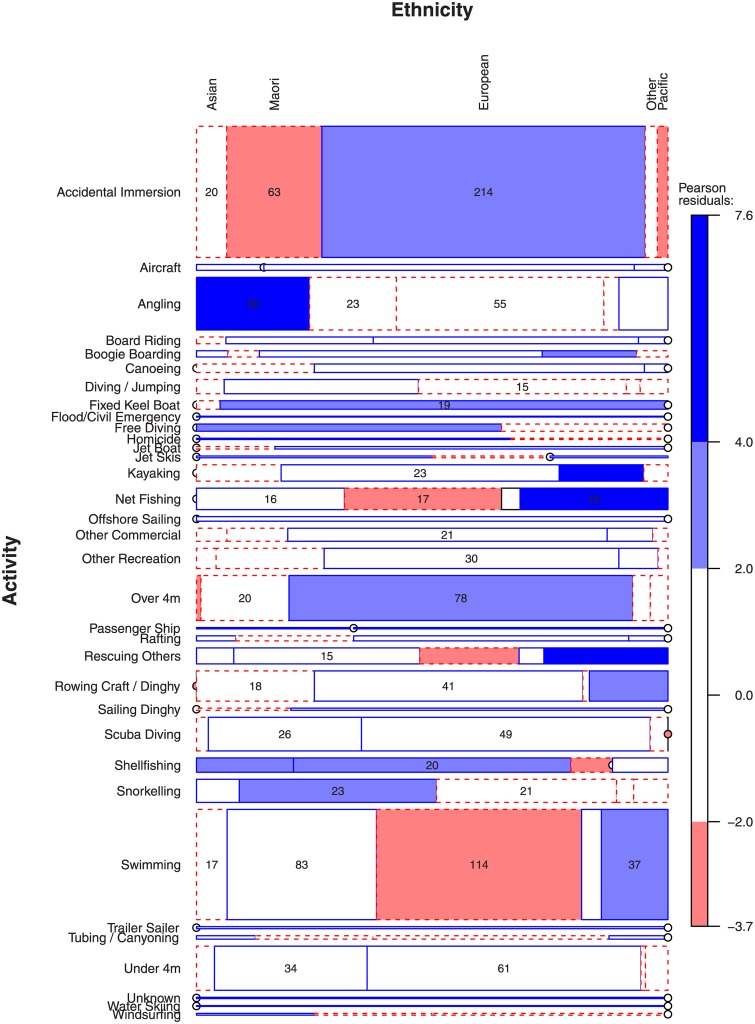
Different ethnicities drown while engaged in different activities.

Different ethnicities drown while engaged in different activities (Fig 3). NZ Europeans are over-represented in Accidental Immersions and Powered Boats Over 4m and under-represented in Swimming. Asians are strongly over-represented in drownings following Angling. Pacific people are strongly over-represented in drownings following Net Fishing and Saving Others. Māori are under-represented in Accidental Immersions cases and over-represented in Snorkeling.

**Fig 4:**
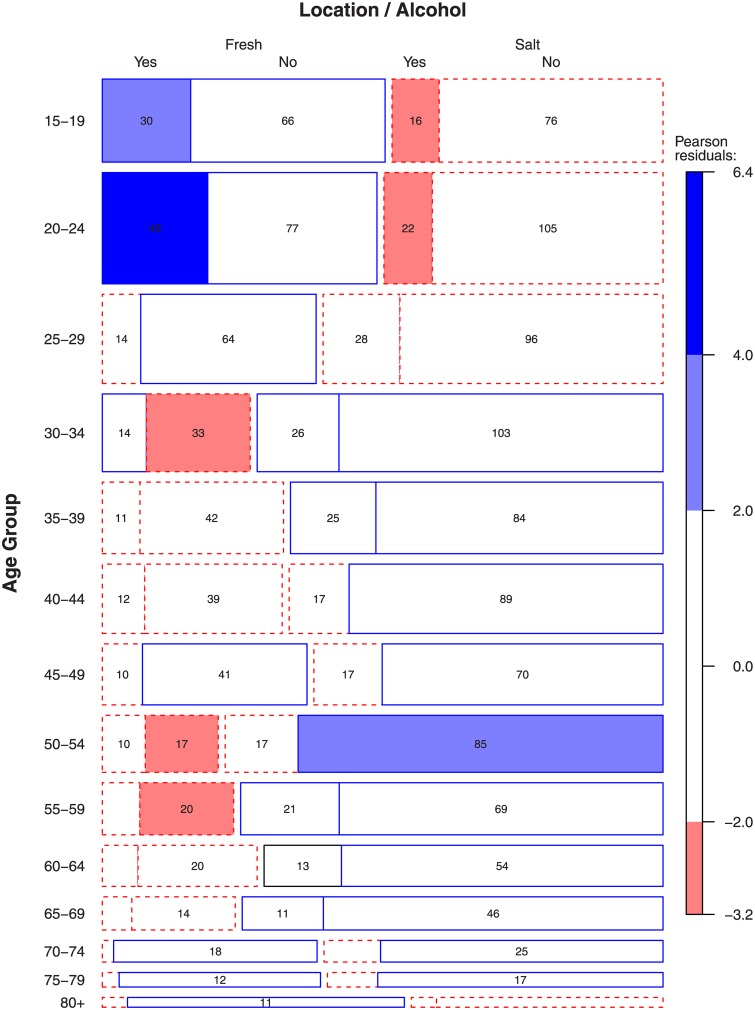
Age group affects the location (freshwater vs. saltwater) that precedes drowning and whether alcohol was involved.

The number of drownings in swimming pools was small for all adults so we focused on open water locations. The most prevalent age group for drownings involving alcohol in males is 20–24 years old. In the younger age groups (less than 25 years), roughly half of the drownings occurred in fresh water and half in salt water (Fig 4). Interestingly, a large proportion of the fresh water drownings involved alcohol, whereas those in salt water did not. In older age groups (more than 50 years) there were more drownings in salt water than fresh water but none of the other age groups had a significant association between alcohol usage and water type. Apparently, the location of drowning (and therefore also the associated activity) seems to influence the relative likelihood of alcohol use.

**Fig 5:**
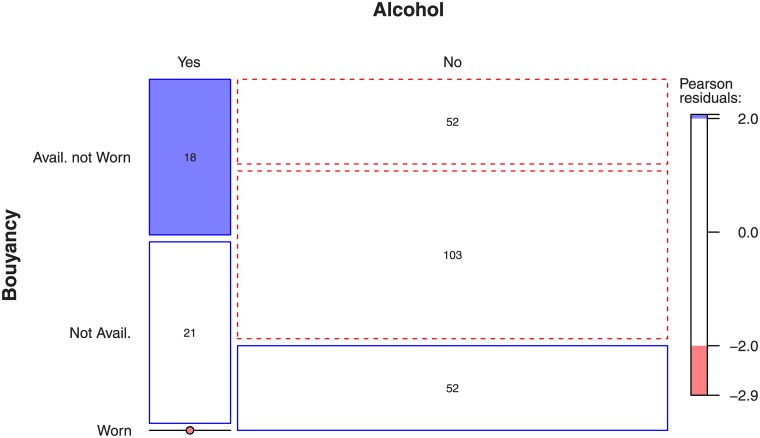
The association of wearing life jackets with alcohol consumption where Power Boats were involved in drowning.

Of the 77 cases that involved alcohol and boating, a buoyancy aid was worn in only one of those cases. In 26 of those cases a buoyancy aid was ‘Available But Not Worn’. In Powered Boats, the proportion of cases where a buoyancy aid was ‘Available But Not Worn’ was higher (18 of 39). None of the alcohol related drowning victims from Powered Boats had worn a buoyancy aid. Presumably boaters believe that they are safer in powered boats and choose not to wear life jackets (Fig 5). When alcohol was involved there were proportionally more incidences where a life jacket was available but not worn and proportionally less incidences where a life jacket was worn. However, the precise influence of ‘alcohol use’ by the drowned individual is not clear due to the absence of blood-alcohol data recorded in Drownbase.

## Discussion

Whilst numerous explanations have been offered concerning increased risk of drowning in adult males they are often proposed independently, yet it is highly likely that they are related. In the analysis presented here we used conditional frequency tables (displayed as mosaic plots) to begin to uncover how these factors act in combination to influence drowning risk. The distribution of residual statistics can be used to identify where features of the data are significantly different from that which might be expected. For example, the age of victims and the activity that they were performing were clearly related in New Zealand drownings. Younger males (15–24 years old) were identified as more likely to be involved in water sports or recreational activities like swimming, whereas older males (older than 55 years) were more often drowned whilst out boating. Whilst such relationships may seem intuitive based upon different physical capabilities and levels of financial independence, to our knowledge this is the first time that data has been mined specifically to identify such features. We recommend that epidemiology researchers consider using conditional tables (amongst other analysis tools) to provide a more thorough analysis of risk factors influencing drowning.

On the basis of these findings concerning males there are numerous potential intervention strategies that may warrant consideration. For example, the Water Safety sector might provide educational campaigns targeted at certain age group of males that explain the risks and suitable safety precautions associated with Water Sport / Recreation activities. Young males need to realistically evaluate their swimming capabilities within open water environments that are not typically supervised (e.g., lakes, rivers, some beaches). It might be prudent to disseminate information about the hidden risks associated with recreation in open water (e.g., cold shock, rip tides, occluded objects in the water, etc.). Future research should consider how and why accidental immersion and alcohol use are connected (i.e., impaired decision making, perception, increased risk-taking) and what appropriate precautions (i.e., always wear lifejacket) are necessary.

A high proportion of 30–39 year old males in New Zealand drown during underwater activities (e.g., snorkeling, diving). In snorkeling inexperienced divers tend to die on or near the water surface, whereas the experienced tend to die after prolonged breath-hold dives, possibly due to the practice of hyperventilation prior to submersion [23]. Pre-dive medical assessments should be undertaken for people with a history of cardiac or respiratory disease.

Over the last 30 years, NZ Europeans in the 65–74 years age group were heavily represented in drowning statistics (Fig 3). In older age groups there were more drownings in salt water than fresh water. Older men (aged +55 years old) had a high incidence of drowning while boating (non-powered boat and powered boat). This feature of the data was particularly prominent in cases from 2012, where 20 deaths (22% of 93 deaths in total) resulting from powered boating represented a 150% increase on the five-year average.

It is possible that visitors to New Zealand and those with limited aquatic experience are not knowledgeable enough about the local geography and conditions. As such it may be prudent for water safety organizations and aquatic leisure facilities to provide better information about the risk of recreational boating for those not familiar with New Zealand’s conditions. Preventative strategies that reach ethnic minorities amongst the fishing community would also appear to be worth further consideration (Fig 3). In New Zealand, people may engage in risky aquatic activities (e.g., diving for food) that have some cultural significance. As such education and diving training would seem appropriate measures to reduce risk whilst respectful of cultural practices.

Howland et al. (1990) reported that men who drank and went power boating were less likely to wear life jackets than those who did not drink (9% vs. 30%). The mortality data from New Zealand provides some support for these self-reported habits in the USA. In New Zealand, firmer regulations may be required for recreational boating use in areas of known high drowning risk (e.g., coastlines, harbours, etc.). There was a low incidence of wearing a life jacket while consuming alcohol across all types of boating but the availability of life jackets appears to have been lower, so people may have not worn them because they were not available. The availability of buoyancy aids and subsequent patterns of usage continues to be an area of concern for boat-users in New Zealand.

Our analysis of Drownbase has unearthed some interesting data related to male drownings in New Zealand. Whilst some of these findings are common to other countries (as identified in the literature review), there also appears to be New Zealand specific factors that relate to an increased risk of drowning amongst adult males (in comparison to 0–5 years old children). For example, deaths from recreational boating are higher in NZ than other developed countries. Between 1980 and 1994 boating was identified as the main activity accounting for 622 deaths (Langley, et al., 2001). This equates to ~41 p.a. and 1.3 per 100,000 population. In Australia (2002–2007) there were 151 watercraft related drownings (Franklin, et al., 2010), which equates to 0.15 per 100,000. In the United States, Washington State had a population of 4–5.5M for 1980–1995, which is similar to NZ, and yet they only had 120 boating fatalities (Quan & Cummings, 2003), 20% of those reported in NZ. Elsewhere in the US, Maine had 82 boating related deaths between 2000 and 2007 (Gilchrist, et al., 2008), which equates to 0.8 per 100,000. In New York State from 1988 to 1994, there were 216 watercraft related drownings (Browne, et al., 2003b), equating to 0.17 per 100,000. In England and Wales (1982–1987) there were 36 boating related drownings (including sailing, surfboarding and wind surfing) (Avery, et al., 1990), which equates to 0.01 per 100,000. In some of these areas people don’t have access to water or boating isn’t a part of life, so the incidence of boating related drownings is likely to be lower. In other areas, such as Washington State, 26% of residents own a boat and about 40% of those own a powerboat (National Recreational Boating Survey, 2011). About 35% of residents participated in some form of boating in the previous year. One difference between places like Washington State and NZ is the boating laws, which are less strict in NZ.

Another difference between NZ and other developed countries is the high incidence of deaths from rock fishing, diving/snorkeling; practices that are strongly linked to ethnic groups common to NZ (Asian and Māori). Hence international drowning prevention strategies may not be as effective in New Zealand as they are in other countries.

## Limitations

A large portion of the literature review contained epidemiological studies which tend to be descriptive. A greater range of more sensitive research methodologies are required to better understand the reasons why so many more males than females drown. The Drownbase information is valuable in monitoring trends and highlighting potential prevention strategies, however in some cases more specific data, such as blood alcohol content, is required before formal recommendations can be made.

## Conclusions

New Zealanders of different ages and ethnicities typically drown in different locations, doing different activities. As such, carefully considered and targeted intervention strategies (as opposed to a blanket, ‘one-size-fits-all’ approach) are required to reduce drowning risk in this country. For example, in New Zealand firmer regulations may be required for recreational boating use in areas of known high drowning risk (e.g., coastlines, harbours, etc.). There was a low incidence of wearing a life jacket while consuming alcohol across all types of boating but the availability of life jackets appears to have been lower, so people may have not worn them because they were not available.

It is possible that visitors to New Zealand and those with limited aquatic experience are not knowledgeable enough about the local geography and conditions. As such it may be prudent for Water Safety organisations to provide better information about the risk of recreational boating for those not familiar with New Zealand’s conditions. Preventative strategies that reach ethnic minorities amongst the fishing community would also appear to be worth further consideration. Finally, when considering drowning intervention strategies used internationally, their relative effectiveness must be weighed up against some of the risk factors documented in this report that are specific to New Zealand.
